# Differences in the effects of orthodontic treatment on airway-craniocervical functional environment in adult and adolescent patients with skeletal class II high-angle: a retrospective pilot study

**DOI:** 10.1186/s12903-023-03328-w

**Published:** 2023-08-29

**Authors:** Yiyang Shen, Xin Li, Xiaoyan Feng, Lan Yu, Luxi Weng, Chenxing Zhang, Yufeng Shang, Jun Lin

**Affiliations:** 1 Department of Stomatology, The First Affiliated Hospital, College of Medicine, Zhejiang University, Hangzhou, Zhejiang China; 2 Key Laboratory of Oral Biomedical Research of Zhejiang Province, Zhejiang University School of Stomatology, Hangzhou, Zhejiang China; 3 The Affiliated Lihuili Hospital, Ningbo University, Ningbo, Zhejiang China; 4 Department of Stomatology, Hangzhou Geriatric Hospital, Hangzhou, Zhejiang China

**Keywords:** Skeletal class II high-angle, Adolescent, Adult, Craniocervical posture, Upper airway

## Abstract

**Introduction:**

This retrospective cohort study aimed to compare the change in upper airway and craniocervical posture after orthodontic treatment between adolescent and adult patients with Class II high-angle malocclusion.

**Methods:**

A total of 12 adolescent (mean ± standard deviation age = 13.0 ± 2.0 years) and 12 adult patients with Class II high-angle malocclusion (mean ± standard deviation age = 23.7 ± 6.4 years) were selected in this study. The lateral cephalograms and cone beam computed tomography images of adolescent and adult patients were taken before and after treatment, which can be employed to evaluate the variables of craniofacial morphology, upper airway, and craniocervical posture through paired t tests, respectively. An independent sample t test was performed to observe the differences between two groups after orthodontic intervention. For adults and adolescents, the correlation between craniofacial morphology, upper airway, and craniocervical posture was determined through Pearson correlation analysis.

**Results:**

In all subjects, the improvements in vertical and sagittal facial morphology after treatment were observed. Anterior and inferior movements of the hyoid bone, an increase of upper airway dimension, posterior tipping of the head and a reduction of cervical inclination in the lower and middle segments post-treatment were identified in adolescence (*P* < 0.05). Adults displayed anterior movements of the hyoid bone, whereas no significant difference was observed in upper airway dimension and craniocervical posture (*P* < 0.05). Notable differences were identified in the change of hyoid position and airway volume between two groups (*P* > 0.05). Mandibular plane inclination, growth pattern, occlusal plane inclination, and chin position were all significantly correlated with craniocervical posture in adolescent patients. Besides, the mandibular growth pattern and chin position in adult patients were significantly correlated with craniocervical posture (*P* < 0.05).

**Conclusions:**

Orthodontic treatment is capable of enhancing the facial profile of patients with skeletal class II high-angle while improving their upper airway morphology and craniocervical posture, where adolescents and adults differ substantially in that the former exhibit a more favorable alteration in the airway-craniocervical functional environment.

## Background

Skeletal Class II high-angle malocclusion refers to one of the most challenging malocclusions in orthodontics; it exhibits sagittal underdevelopment and vertical overdevelopment of the mandible [1, 2]. Under the effect of this malocclusion, patients tend to develop poor lateral appearances and restricted airways [3, 4]. Forward mandibular rotation should be incorporated into any treatment plan to tackle down skeletal deformity to address the plethora of difficulties correlated with hyperdivergent Class II patients [5, 6]. While skeletal effect of orthodontic treatment differs in growth potential [6, 7]. The above-described forward mandibular rotation further stimulates the anterior growth of the mandible of a number of growing patients, such that the skeletal facial pattern is enhanced [8]. However, the mentioned therapeutic effects are limited in adult patients.

Patients subjected to skeletal class II high angles will face a higher risk of obstructive sleep apnea hypopnea syndrome (OSAHS) [9]. OSHAS is capable of reducing patients' sleep quality at night, adversely affecting patients' daily work and life, and elevating the risk of serious systemic diseases (e.g., hypertension, diabetes, and coronary heart disease) in adults [10–12]. As revealed by recent research, OSHAS patients are getting younger, i.e., an increasing number of adolescents are subjected to narrowed airways [13–15]. Compared with adults, airways of adolescent patients were narrower and shorter [16] and the larynx is softer and more pliable, which increases the risk of airway obstruction [17]. Adolescents with constricted airways are more likely to be subjected to stunted height and weight growth, cognitive and attention deficit or hyperactivity, poor academic performance, as well as emotional instability [18–22]. Thus, it is crucial to undertake upper airway monitoring and orthodontic treatment on patients with Class II high-angle malocclusion, particularly adolescents.

Craniocervical posture refers to a condition that preserves the relative stability of the craniofacial and cervical regions in the external and internal environment, and it frequently reflects the outcome of the coordination of gravity and functional demands [23]. Craniocervical posture is linked to both sagittal and vertical skeletal facial morphology. Actually, patients in skeletal Class II show a more lordotic curve of the spine and a larger extension of the head than those in Class III, and as a result, exhibit a significantly greater craniocervical angle [23–25]. Additionally, the craniocervical angle is notably increased in high-angle patients compared with low-angle populations [23, 26, 27]. To explain this phenomenon, Solow suggests that this stretching of craniocervical posture is a consequence of the patient's effort to obtain a larger airway [28]. In accordance with the above-mentioned theory, several researchers have reported significant changes in craniocervical posture in response to the relief of airway obstruction [29–31]. In contrast, modifications to craniocervical posture can affect growth patterns. Longitudinal research has suggested that people with smaller craniocervical angles are prone to horizontal growth pattern, whereas those with larger craniocervical angles are prone to vertical growth pattern [32, 33]. However, the effect of orthodontic therapy on changes craniocervical posture in class II high-angle patients has been rarely examined, particularly the comparison of the disparities between the two populations, adolescents and adults.

Since the improvement of craniofacial morphology affects airway and craniocervical posture, a hypothesis was proposed that orthodontic treatment will lead to a comparable improvement in the airway and craniocervical posture in patients with skeletal class II high-angle, and this improvement will become more prominent in adolescents. Accordingly, this retrospective cohort research aimed to determine in patients with skeletal class II high-angle malocclusion: (1) the effects of orthodontic treatment on upper airway and craniocervical posture in adolescents and adults, respectively; (2) the differences in the effects of treatment on airway and craniocervical posture in the two patient groups; (3) the correlation between craniofacial morphology, upper airway and craniocervical posture in adolescents and adults, respectively.

## Methods

This retrospective study gained approval from the Clinical Research Ethics Committee of the First Affiliated Hospital, College of Medicine, Zhejiang University (protocol code: (2021) IIT (171) and date of approval: 10 March 2021). All subjects were informed of the purpose of this study and gave informed consent prior to the study. A total of 12 adult and 12 adolescent participants were selected from all patients requiring orthodontic treatment from January 2016 to July 2021 in the Department of Stomatology, The First Affiliated Hospital of Zhejiang University Medical College. The inclusion criteria are elucidated as follows: (1) adult patients (age ≥ 18 years old) and adolescent patients (aged from 11 to 17 years old); (2) skeletal Class II malocclusion (ANB angle ≥ 4°) and high-angle pattern (MP-FH angle ≥ 29°); (3) extraction of two maxillary first premolars and two mandibular second premolars; (4) four micro-implants implanted bilaterally in the maxilla and mandible; (5) available CBCT images before and after treatment. The exclusion criteria are presented as follows: (1) history of orthodontic treatment and/or orthognathic surgery; (2) temporomandibular joint disorders syndrome; (3) history of upper airway surgery; (4) impairment in the lip and/or palate function (e.g., a cleft lip and/or palate).

All patients wore a pre-adjusted edgewise appliance of 0.022-inch slot (3 M Unitek, Monrovia, CA, USA) after their maxillary first premolars and mandibular second premolars were extracted. Four miniscrews (VectorTAS; Ormco, Orange, Calif; length, 8 mm; diameter, 1.4 mm) were implanted bilaterally in the maxilla and mandible between the second premolars and the first molars through the buccal mucosa after local anesthesia by the same orthodontist. A 150 g force load was delivered with an elastic chain four weeks after the placement of microimplant. The treatment objective was Class I canine and molar relationship, and the respective patient's treatment lasted for nearly three years.

CBCT (NewTom VGi, Verona, Italy) was taken in all patients prior to and after orthodontic treatment. During the scan, the patient was instructed to maintain a natural upright head position and maximum intercuspation, with consistent scanning parameters (tube voltage 110 kV, tube current 3.5 mA, exposure time 3.6 s, and definition 0.3 mm). The scanning ranged from the superior orbital edge to the lower mandibular edge. For 3D reconstruction and analysis, all CBCT data were saved in DICOM format and then input into Dolphin Imaging 11.95 software (Chatsworth, Los Angeles, CA, USA).

**Fig. 1:**
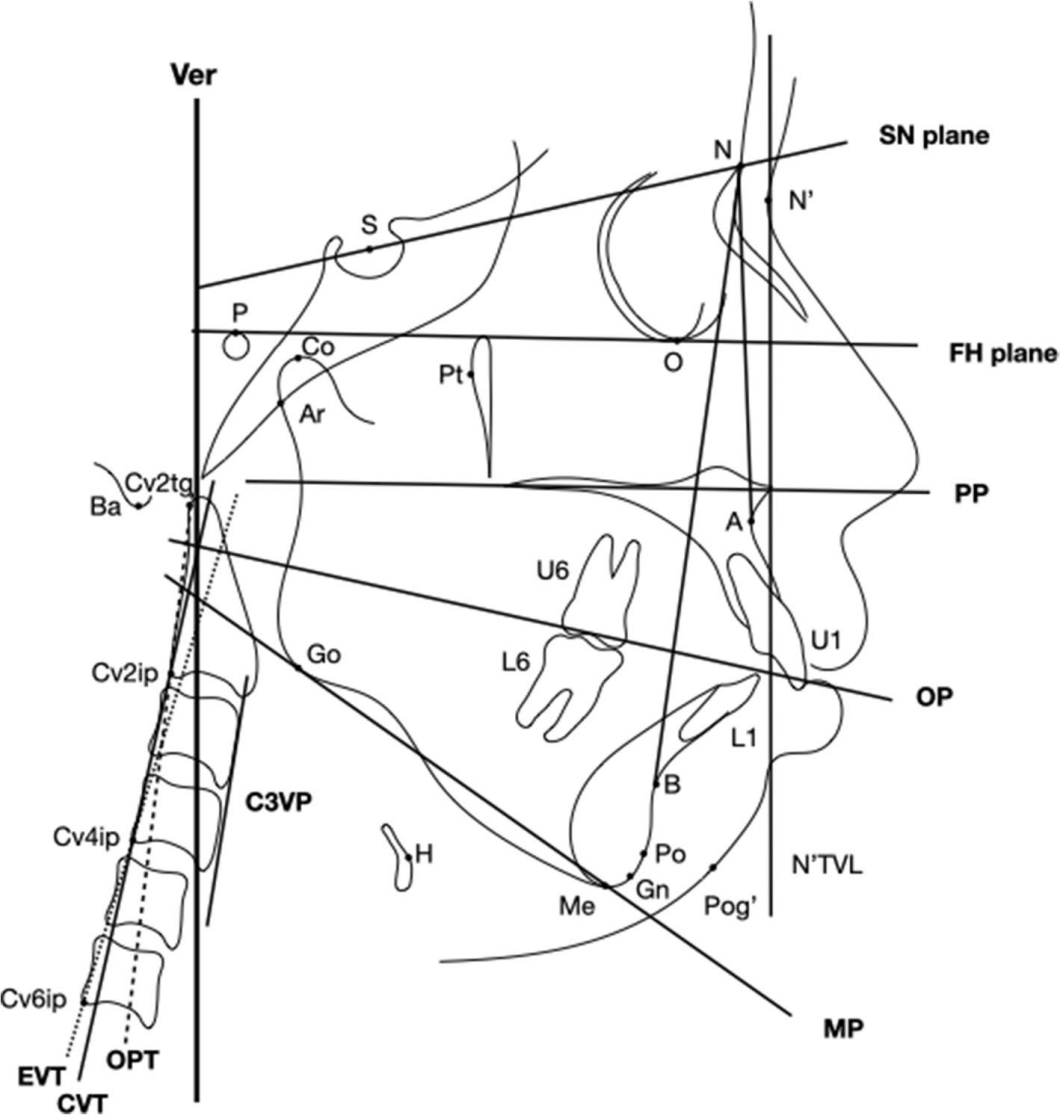
Cephalometric landmarks and measurements identified on lateral cephalometric image. SN plane: the line connecting the point S to N; FH plane: the line connecting the point O to P; PP Plane: the line connecting the point ANS to PNS; OP plane: The line between the midpoint of the upper and mandibular first permanent molar and the midpoint of the upper and mandibular middle incisor; C3VP: The line tangent to the anterior border of the third cervical vertebra; Ver: The gravity-determined vertical line; OPT: The line between the tangent point of the superior, posterior extremity of the odontoid process of the second cervical vertebra (cv2tg) and the most inferior-posterior point of the second cervical vertebra (cv2ip); CVT: The line between the most inferior-posterior point of the second cervical vertebra (cv2ip) and that of the fourth cervical vertebra (cv4ip); EVT: The line between the most inferior-posterior point of the fourth cervical vertebra (cv4ip) and that of the sixth cervical vertebra (cv6ip). For detailed definition of each variable, refer to Table 1

All parameters were measured on the lateral cephalogram from CBCT by projecting the 3D reconstruction image into the midsagittal plane from right to left (Table 1; Fig. 1) to examine dental, skeletal, hyoid position and craniocervical posture indexes.

**Table 1:** Cephalometric landmarks and measurements used in this study

Landmarks	Definition
N	Nasion: the anterior point of the intersection between the nasal and frontal bones
N’	The corresponding point N on the lateral side of the soft tissue
S	Sella: the center of the sella turcica
O	The deepest point on the infra-orbital margin
P	The most superior point of the outline of the external auditory meatus
Ba	The most inferior-posterior point on the margin of the foramen magnum
Pt	Posterior outline of the Pterygo-Maxillary Fissure
A	Subspinale: the most posterior point on the exterior ventral curve of the maxilla
U1	Maxillary central incisor
U6	The near midbuccal tip or sulcal point of the maxillary first molar
Ar	Point of intersection of the inferior cranial base surface and the averaged posterior surfaces of the mandibular condyles
Go	The most posterior-inferior point on the outline of the mandible angle
B	Supraemental: the most posterior point on the bony curvature of the mandible
Po	The most anterior point on the contour of the bony chin
Pog’	The corresponding point Po on the lateral side of the soft tissue
Me	The most inferior point on the outer inferior margin of the mandible
Gn	The most anterior-inferior point on the outline of the bony chin
L1	Mandibular central incisor
L6	The near midbuccal cuspoint of the mandibular first molar
H	Hyoidale: the most superior and anterior point on the body of the hyoid bone
SN Plane	SN Plane: the line connecting the point S to N
PP Plane	PP Plane: the line connecting the point ANS to PNS
NA	the line connecting point A to N
NB	the line connecting point B to N
OP	Occlusal plane: A line between the midpoint of the upper and mandibular first permanent molar and the midpoint of the upper and mandibular middle incisor
MP	Mandibular plane: the line connecting the point Go to Me
FHP	Frankfort plane: the line connecting the point O to P
C3VP	The line tangent to the anterior border of the third cervical vertebra
Ver	The gravity-determined vertical line
OPT	The line between the tangent point of the superior, posterior extremity of the odontoid process of the second cervical vertebra (cv2tg) and the most inferior-posterior point of the second cervical vertebra (cv2ip)
CVT	The line between the most inferior-posterior point of the second cervical vertebra (cv2ip) and that of the fourth cervical vertebra (cv4ip)
EVT	The line between the most inferior-posterior point of the fourth cervical vertebra (cv4ip) and that of the sixth cervical vertebra (cv6ip)
**Measurements**	
Craniofacial morphology	
FMA(°)	The angle between the MP and FHP
ANB(°)	The angle between A and B at N
OP-FH(°)	The angle between the occlusal plane and FHP
U1-SN (°)	The angle between the long axis of U1 and SN plane
U1-NA(mm)	The perpendicular distance between from the tip of maxillary incisor to N-A line
L1-MP (°)	The angle between the long axis of L1 and MP plane
L1-NB(mm)	The perpendicular distance between from the tip of mandibular incisor to N-B line
U6-NA (°)	The angle between the long axis of U6 and N-A line
L6-NB (°)	The angle between the long axis of L6 and N-B line
Sum(°)	The sum of Jarabak angles (∠N-S-Ar, ∠S-Ar-Go and ∠Ar-Go-Me)
NBa-PtGn(mm)	The angle formed by lines NBa and line PtGn
Pog’-N’TVL (mm)	Linear distance from Pog’ to N’ true vertical line
Upper airway	
Hyoid position	
H-MP(mm)	The perpendicular distance from H to MP
H-FHP(mm)	The perpendicular distance from H to FH plane
H-C3VP(mm)	The perpendicular distance from H to C3VP plane
Craniocervical posture	
Cervical inclination	
CVT/EVT(°)	The angle between the CVT and EVT
OPT/Ver(°)	The angle between OPT and the vertical line
CVT/Ver(°)	The angle between CVT and the vertical line
EVT/Ver(°)	The angle between EVT and the vertical line
Craniofacial inclination	
SN/Ver(°)	The angle between SN plane and the vertical line
PP/Ver(°)	The angle between PP plane and the vertical line
Craniocervical inclination	
SN-CVT(°)	The angle between SN plane and CVT
SN-OPT(°)	The angle between SN plane and OPT

**Fig. 2:**
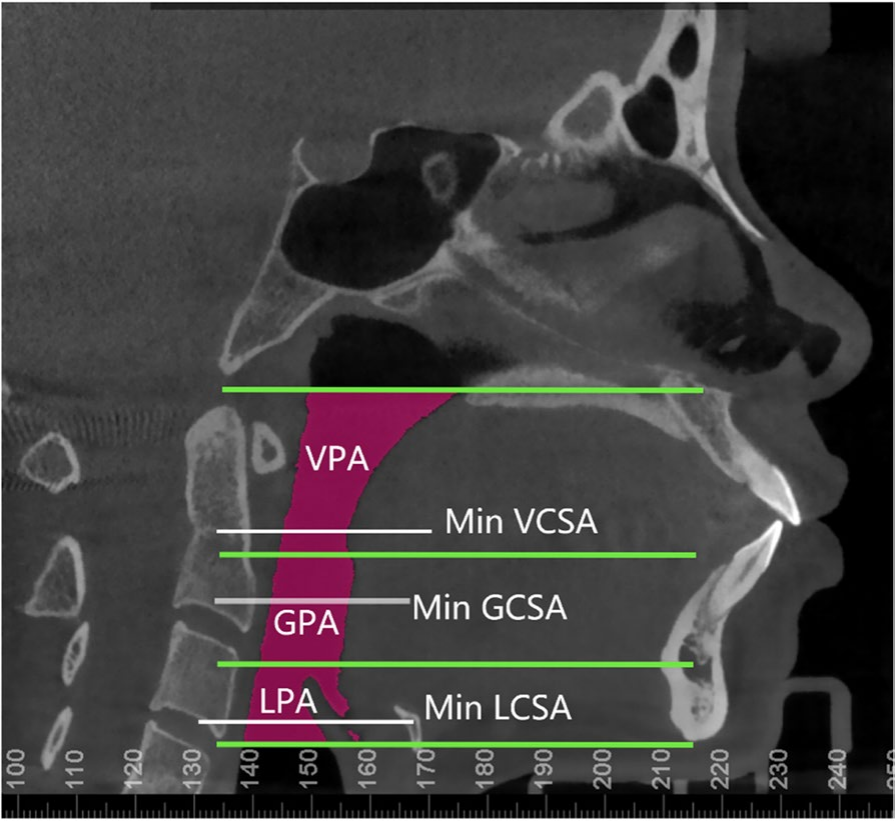
Segments of the upper airway on midsagittal CBCT image using Dolphin Imaging 11.95 software (Chatsworth, Los Angeles, CA, USA). Velopharynx airway (VPA): from the plane of ANS-PNS to the plane across the most posteroinferior point of the uvula; Glossopharynx airway (GPA): from the plane across the most posteroinferior point of the uvula to the plane across the most superior point of the epiglottis. Laryngopharynx airway (LPA): from the plane across the most superior point of the epiglottis to the plane across point of the epiglottic vallecula. Minimum cross-sectional area of each upper airway segment (Min VCSA, Min GCSA and Min LCSA) was automatically identified and measured by the Dolphin software

The airway dimensions were examined before and after treatment based on Dolphin Imaging software. All images were standardized in orientation with the PP plane parallel to the horizontal plane. All planes defining the upper airway boundary were parallel to the PP plane. The upper airway was manually divided into three midsagittal parts (i.e., velopharynx airway (VPA), glossopharynx airway (GPA) and laryngopharynx airway (LPA)) (Fig. 2). The volume and minimum areas of VPA, GPA and LPA were obtained automatically using Dolphin software after the boundaries were set.

To be specific, SPSS 26 software (IBM Corp., Armonk, NY, USA) was employed. Whether the data followed a normal distribution was determined through the Kolmogorov–Smirnov test. A comparison was drawn between pretreatment and posttreatment outcome variables through Wilcoxon signed rank test for nonnormally distributed variables and through paired t test in terms of normally distributed variables. Moreover, adolescent and adult outcome factors were compared through unpaired t test for normally distributed variables and Wilcoxon signed rank test in terms of non-normally distributed variables. Furthermore, correlation analysis for the respective measurement index was conducted prior to treatment. Items that followed a normal distribution were measured through Pearson correlation analysis, while those that were not normally distributed were examined through Spearman rank correlation analysis. The bilateral test level was set at α = 0.05, and *p* < 0.05 indicated a difference that achieved statistical significance.

## Result

A total of 31 patients who conformed to the inclusion criteria were selected from over 400 orthodontic treatment recordings. Six of them were eliminated due to temporomandibular joint problems (*n* = 5) and prior to orthodontic therapy (*n* = 2). Lastly, 24 patients' records were analyzed, comprising 12 adults (mean ± standard deviation age = 23.7 ± 6.4 years) and 12 adolescents (mean ± standard deviation age = 13.0 ± 2.0 years). All patients fulfilled the treatment aim of a Class I canine and/or molar relationship with an improved facial profile.

First, the 13 craniofacial morphology indices between the pre-treatment skeletal Class II high-angle adolescent and adult patients are not significantly different (Table 2).

**Table 2:** Comparison of dental and skeletal variables before orthodontic treatment between adolescents and adults

Variables	Adolescents	Adults	*P* value
FMA(°)	35.21 ± 4.55	32.35 ± 3.73	0.107
SNA(°)	80.40 ± 3.19	81.50 ± 3.03	0.397
SNB(°)	74.35 ± 2.76	74.37 ± 3.175	0.988
ANB(°)	6.04 ± 1.22	7.07 ± 1.93	0.136
U1-SN(°)	105.73 ± 4.95	102.30 ± 8.21	0.227
U1-NA(mm)	6.02 ± 1.80	4.67 ± 2.60	0.153
L1-MP(°)	95.01 ± 5.92	97.61 ± 6.21	0.306
L1-NB(mm)	9.18 ± 2.11	9.09 ± 2.22	0.921
U6-NA(mm)	24.70 ± 1.76	25.15 ± 2.82	0.650
L6-NB(mm)	16.32 ± 2.42	14.77 ± 3.02	0.179
S-Ar-Go(°)	154.81 ± 6.35	152.19 ± 7.90	0.380
Sum(°)	402.89 ± 2.71	401.34 ± 5.36	0.381
NBa-PtGn(mm)	79.15 ± 1.91	80.29 ± 4.63	0.440
Pog’-N’TVL(mm)	-7.92 ± 3.90	-6.95 ± 6.88	0.673
OP-FH(°)	13.37 ± 4.03	12.25 ± 3.33	0.465

Table 3 lists the parameter of the craniofacial morphology, upper airway, and craniocervical posture in adolescent patients before and after treatment. After treatment, OP-FH, FMA and ANB were dramatically decreased by 1.99°, 2.60°, and 2.55° compared with baseline measurements, thus suggesting a counterclockwise rotation of the occlusal plane and the mandible, respectively. In addition, the Sum angle significantly declined by 2.56°, representing a higher propensity for horizontal growth of the mandible. The NBa-PtGn and Pog’-N’TVL were significantly increased by 1.28 mm and 4.38 mm, thus suggesting the forward movement of the chin. For upper airway indicators, H-MP, H-FHP, and H-C3VP were significantly increased, indicating a forward and downward position of the hyoid bone. Moreover, the volume (VPA, GPA and LPA) and minimum cross-sectional area (Min VCSA, Min GCSA, and Min LCSA) of the respective upper airway segment were notably increased after treatment. For changes in craniocervical posture, the values of OPT/Ver, CVT/Ver and EVT/Ver were markedly elevated by 2.83°, 3.58° and 5.00°, respectively. All the two craniofacial inclination angles (i.e., SN/Ver and PP/Ver) were significantly increased by 1.83° and 3.50°. Furthermore, the craniocervical angle SN-CVT was notably decreased by 1.67° after treatment.

**Table 3:** Comparison of all variables before and after orthodontic treatment in adolescent patients

Variables	Pretreatment	Posttreatment	*P* value
Craniofacial morphology			
FMA(°)	35.21 ± 4.55	32.61 ± 4.80	< 0.001**
SNA(°)	80.40 ± 3.19	80.23 ± 2.33	0.795
SNB(°)	74.35 ± 2.76	76.75 ± 2.95	0.005*
ANB(°)	6.04 ± 1.22	3.49 ± 1.64	< 0.001**
OP-FH(°)	13.37 ± 4.03	11.38 ± 4.44	0.027*
Sum(°)	402.89 ± 2.71	400.33 ± 3.05	< 0.001**
NBa-PtGn(mm)	79.15 ± 1.91	80.43 ± 1.15	0.049*
Pog’-N’TVL(mm)	-7.92 ± 3.90	-3.54 ± 4.37	0.014*
Upper airway			
Hyoid position			
H-MP(mm)	10.19 ± 3.90	13.39 ± 4.75	0.022*
H-FHP(mm)	78.66 ± 5.19	85.84 ± 6.73	0.001**
H-C3VP(mm)	27.04 ± 2.58	30.61 ± 4.62	0.025*
Upper airway dimensions			
VPA(mm^3^)	7234.99 ± 4002.62	10,822.18 ± 2722.24	0.003**
GPA(mm^3^)	4057.67 ± 2715.38	6793.93 ± 2040.76	0.007**
LPA(mm^3^)	2493.55 ± 1478.26	4726.30 ± 2578.45	0.003**
Min VCSA(mm^2^)	67.58 ± 47.57	105.59 ± 55.10	0.059
Min GCSA(mm^2^)	70.91 ± 29.01	107.13 ± 28.58	0.002**
Min LCSA(mm^2^)	66.50 ± 22.23	105.26 ± 38.57	0.015*
Craniocervical posture			
Cervical inclination			
CVT/EVT(°)	2.50 ± 7.32	0.92 ± 6.10	0.438
OPT/Ver(°)	-6.50 ± 5.27	-3.67 ± 4.66	0.017*
CVT/Ver(°)	-10.50 ± 4.83	-6.92 ± 4.76	0.003**
EVT/Ver(°)	-12.83 ± 6.77	-7.83 ± 6.35	0.011*
Craniofacial inclination			
SN/Ver(°)	96.50 ± 5.07	98.33 ± 4.58	0.038*
PP/Ver(°)	86.08 ± 4.48	89.58 ± 3.37	0.004**
Craniocervical inclination			
SN-CVT(°)	106.92 ± 6.68	105.25 ± 6.15	0.009**
SN-OPT(°)	103.08 ± 5.26	102.00 ± 4.09	0.314

^*^Represents the variables in the adolescents versus adults with *p*<0.05

^**^Represents the variables in the adolescents versus adults with *p*<0.01

Table 4 lists the same outcome variables in adult patients before and after treatment. After treatment, the OP-FH, FMA, ANB and Sum values in the adult group were also significantly decreased by 1.41°, 2.27°, 1.60°, and 2.05°, respectively, indicating a counterclockwise rotation of the occlusal plane and the mandible in adults after the orthodontic intervention. Moreover, NBa-PtGn and Pog’-N’TVL were significantly increased by 1.39 mm and 3.61 mm, respectively, illustrating the anterior displacement of the chin in adult patients. With respect to the upper airway variables, only H-C3VP exhibited a statistically significant increase, suggesting a forward position of the hyoid bone after our orthodontic intervention. However, no differences with statistical significance were observed in upper airway dimensions and craniocervical posture.

**Table 4:** Comparison of all variables before and after orthodontic treatment in adult patients

Variables	Pretreatment	Posttreatment	*P* value
Craniofacial morphology			
FMA(°)	32.35 ± 3.73	30.08 ± 3.76	< 0.001**
SNA(°)	81.50 ± 3.03	80.59 ± 3.28	0.114
SNB(°)	74.37 ± 3.17	75.12 ± 3.12	0.170
ANB(°)	7.07 ± 1.93	5.47 ± 1.83	< 0.001**
OP-FH(°)	12.25 ± 3.33	10.84 ± 3.71	0.008**
Sum(°)	401.34 ± 5.36	399.29 ± 4.78	< 0.001**
NBa-PtGn(mm)	80.29 ± 4.63	81.68 ± 3.59	0.019*
Pog’-N’TVL(mm)	-6.95 ± 6.88	-3.34 ± 5.39	0.001**
Upper airway			
Hyoid position			
H-MP(mm)	11.27 ± 4.10	10.77 ± 3.95	0.666
H-FHP(mm)	83.51 ± 7.58	85.49 ± 6.64	0.439
H-C3VP(mm)	31.14 ± 3.54	32.51 ± 4.16	0.002**
Upper airway dimensions			
VPA(mm^3^)	11,449.38 ± 3676.50	11,297.13 ± 5654.42	0.914
GPA(mm^3^)	9258.60 ± 4722.74	8060.58 ± 4350.84	0.378
LPA(mm^3^)	4545.80 ± 1691.65	4795.79 ± 2198.18	0.570
Min VCSA(mm^2^)	143.65 ± 114.15	134.32 ± 110.49	0.646
Min GCSA(mm^2^)	155.18 ± 86.19	144.34 ± 110.14	0.745
Min LCSA(mm^2^)	120.41 ± 71.46	119.40 ± 71.70	0.968
Craniocervical posture			
Cervical inclination			
CVT/EVT(°)	1.08 ± 4.81	0.67 ± 4.76	0.629
OPT/Ver(°)	-1.50 ± 3.37	0.67 ± 4.76	0.267
CVT/Ver(°)	-8.92 ± 3.80	-7.42 ± 4.60	0.149
EVT/Ver(°)	-10.00 ± 7.12	-8.08 ± 6.40	0.087
Craniofacial inclination			
SN/Ver(°)	100.67 ± 4.40	101.75 ± 3.22	0.254
PP/Ver(°)	88.75 ± 4.00	89.67 ± 1.72	0.347
Craniocervical inclination			
SN-CVT(°)	109.58 ± 5.95	109.17 ± 6.41	0.708
SN-OPT(°)	102.17 ± 5.844	102.50 ± 5.71	0.723

^*^Represents the variables in the adolescents versus adults with *p*<0.05

^**^Represents the variables in the adolescents versus adults with *p*<0.01

To conduct an in-depth investigation of the differences in the treatment of upper airway and craniocervical posture between orthodontic procedures in adult and adolescent patients with skeletal class II high angles, we evaluated seventeen variables, as depicted in Table 5. The values of ΔH-MP in adult patients were considerably lower than those in adolescent patients, indicating that the location of the hyoid bone was elevated in adult patients following therapy. Moreover, the ΔVPA, ΔGPA, and ΔLPA of adult patients were significantly lower than those of adolescent patients, suggesting that adolescent patients had more pronouncedly improved upper airway dimensions after orthodontic intervention. Nevertheless, no significant differences were observed in cervical, craniofacial and craniocervical indices.

**Table 5:** Comparison of effects post treatment between adolescents and adults

Variables	Adolescents	Adults	*P* value
Upper airway			
Hyoid position			
ΔH-MP(mm)	3.20 ± 4.15	-0.50 ± 3.94	0.035*
ΔH-FHP(mm)	7.18 ± 5.80	1.98 ± 8.53	0.094
ΔH-C3VP(mm)	3.57 ± 4.78	1.37 ± 1.17	0.135
Upper airway dimensions			
ΔVPA(mm^3^)	3587.19 ± 3244.99	-152.25 ± 4790.28	0.036*
ΔGPA(mm^3^)	2736.27 ± 2844.27	-1198.03 ± 4517.02	0.018*
ΔLPA(mm^3^)	2232.75 ± 2070.24	249.99 ± 1479.03	0.013*
ΔMin VCSA(mm^2^)	38.01 ± 62.65	-9.33 ± 68.38	0.091
ΔMin GCSA(mm^2^)	36.23 ± 29.93	-10.83 ± 112.6	0.176
ΔMin LCSA(mm^2^)	38.76 ± 46.71	-1.01 ± 85.58	0.212
Craniocervical posture			
Cervical inclination			
ΔCVT/EVT(°)	-1.58 ± 6.82	-0.42 ± 2.91	0.591
ΔOPT/Ver(°)	2.83 ± 3.37	0.75 ± 2.26	0.098
ΔCVT/Ver(°)	3.58 ± 3.29	1.50 ± 3.34	0.138
ΔEVT/Ver(°)	5.00 ± 5.63	1.92 ± 3.53	0.122
Craniofacial inclination			
ΔSN/Ver(°)	1.83 ± 2.69	1.08 ± 3.12	0.535
ΔPP/Ver(°)	3.50 ± 3.34	0.92 ± 3.23	0.067
Craniocervical inclination			
ΔSN-CVT(°)	-1.67 ± 1.83	-0.42 ± 3.75	0.311
ΔSN-OPT(°)	-1.08 ± 3.55	0.33 ± 3.17	0.314

^*^Represents the variables in the adolescents versus adults with *p*<0.05

**Fig. 3:**
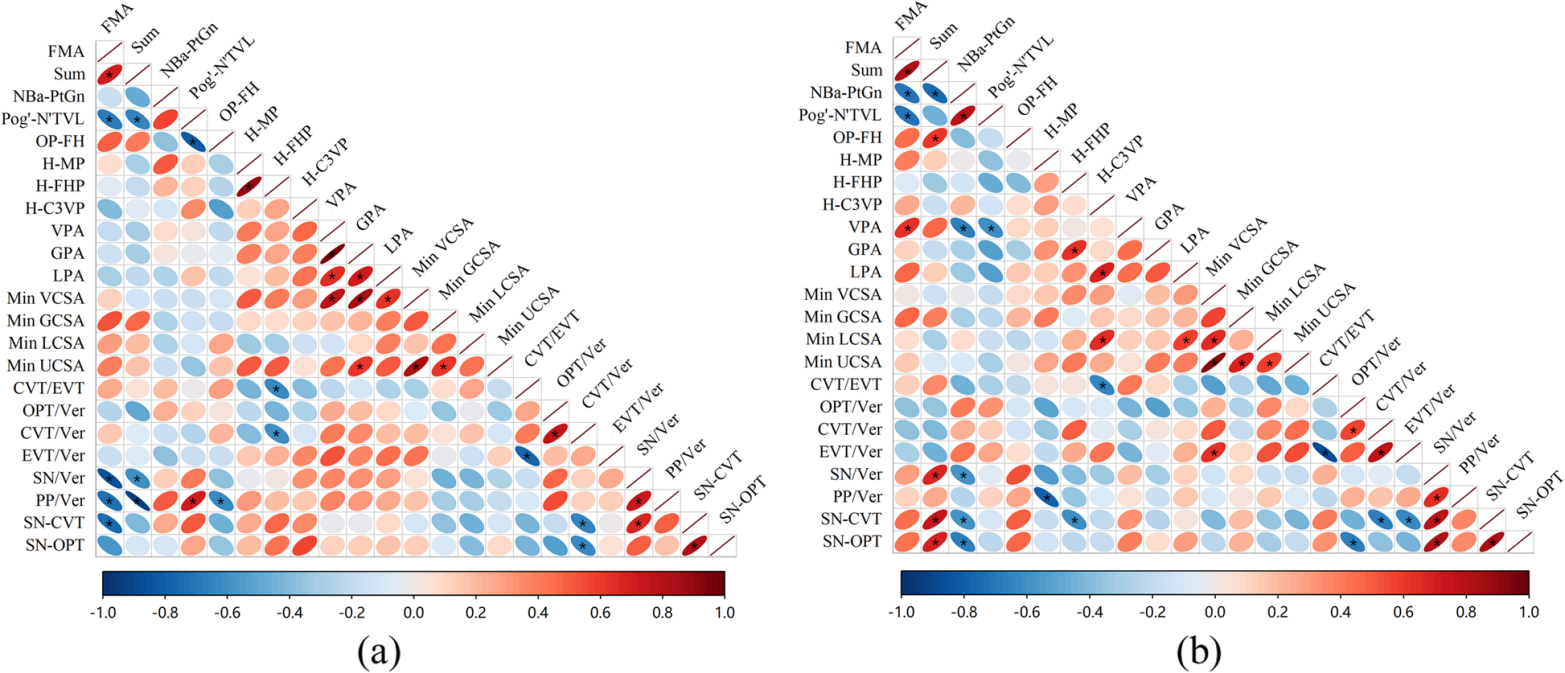
Correlation heatmap of craniofacial morphology, upper airway and craniocervical posture in adolescent (**a**) and adult (**b**) patients. Positive correlation is represented by red ellipses, while negative correlation is represented by blue ellipses, with a deeper hue indicating a stronger correlation. Specifically, the darker the red, the closer the r is to 1, and the darker the blue, the closer the r is to − 1. Similarly, the ellipse's size also fluctuates as a result of variations in the correlation. The closer the r is to 1 (red) or − 1 (blue), the closer the ellipse is to a line, whereas the closer the r is to 0, the closer the ellipse is to a perfect circle. Correlations with significant differences are highlighted in the figure. (*: *p* < 0.05; **: *p* < 0.01;)

Pairwise correlation analysis was conducted on all variables before orthodontic treatment, and correlation heatmaps were generated for adolescents (Fig. 3a) and adults (Fig. 3b). As indicated by the result, cervical variables CVT/EVT and CVT/ver were negatively correlated with H-FHP in adolescent patient. Furthermore, craniofacial angles SN/Ver and PP/Ver were negatively correlated with FMA and Sum, respectively. The value of PP/Ver is also significantly correlated with Pog’-N’TVL and OP-FH. Besides, craniocervical angles SN-CVT was negatively correlated with FMA. While in adult patients, both craniofacial angles SN/Ver and craniocervical angles SN-CVT, SN-OPT were significantly correlated with Sum and NBa-PtGn.

## Discussion

In this study, a major comparison was drawn in terms of the changes of upper airway and craniocervical posture following orthodontic treatment in adult and adolescent patients who were subjected to skeletal class II high-angle malocclusion. Second, the correlation between craniofacial morphology, upper airway, and craniocervical posture was explored. Adult and adolescent patients who underwent orthodontic treatment showed ameliorated upper airway and craniocervical posture. Notably, the teenage patients exhibited more pronounced changes. Furthermore, mandibular plane inclination, growth pattern, occlusal plane inclination, and chin position were markedly correlated with craniocervical posture of adolescent patients. In adult patients, however, only mandibular growth pattern and chin position were significantly correlated with craniocervical posture.

First, the adolescent and adult groups had significantly reduced ANB angles by 2.55° and 1.60° after undergoing treatment. As revealed by this result, the patient's skeletal pattern and profile have been significantly improved through successful orthodontic therapy. This improvement can be manifested in two ways as follows: (1) by employing maximum anchoring in anterior tooth retraction, which can cause alveolar bone remodeling to a certain extent, such that the ANB angle can be improved; (2) by controlling the vertical dimensions of dental arches through micro-implant to reduce the inclination of occlusal plane [34]. For adults, the mandibular plane can be generally rotated counterclockwise by controlling the occlusal plane and acquiring a moderate lingual inclination of the lower anterior teeth, such that the ANB angle is likely to be reduced. The control of the occlusal plane in adolescent patients can contribute to the forward growth pattern of the mandible, such that the SNB and ANB angle can be reduced, and the chin forward can be extended [1].

It is challenging to determine how to counter-rotate the mandible to enhance the poor facial profile of patients with skeletal class II high-angle [5]. The counterclockwise rotation of the occlusal plane can drive the rotation of mandibular plane, as indicated by recent research [1, 34]. Moreover, controlling occlusal plane in growing individuals is frequently accompanied by the downward inclination of mandibular plane [9, 34]. Thus, occlusal plane control takes on a critical significance to the treatment of skeletal class II high-angle malocclusion whether in adult or adolescent patients [35]. Micro-implant anchorage (MIA) technique is capable of preventing the elongation and proximal-central movement of the molar when the extraction space is closed, such that the clockwise rotation effect of the occlusal plane can be avoided [36]. Moreover, a further counterclockwise rotation of the occlusal plane is possible since the micro-implant can act directly on the molar for vertical control [37, 38], suggesting that the MIA technology can contribute to the control of the occlusal plane and even counterrotate the mandible. In this study, micro-implants were adopted to control the occlusal plane, such that the OP-FH was substantially reduced by 1.41° in adult patients and 1.99° in adolescent patients. Furthermore, a considerable counterclockwise rotation of the mandible was achieved by 2.27° and 2.56°, respectively. The above-mentioned result indicated that the skeletal vertical relation was improved significantly in two groups after orthodontic treatment, accompanied by a significant improvement in facial appearance.

It has been widely recognized that the counterclockwise rotation of the mandible may lead to suprahyoid muscle tension and then form an anterosuperior position of the hyoid bone, thus contributing to an enlargement of upper airway dimension [2, 3, 39, 40]. However, the study by Li et al. has suggested that occlusal plane control does not significantly improve the pharyngeal airway dimensions after the treatment of hyperdivergent skeletal Class II malocclusion adult patients [34]. The findings of this study are well consistent with those of Li et al. In the adult group, although the angle of occlusal plane and mandibular plane were lowered, the volume and minimum cross-sectional area of their upper airway did not significantly increase, but the hyoid position was significantly shifted forward.

For adolescent patients, Pavoni C. reported that the adjustment of mandibular retrusion with functional appliances in Class II malocclusion adolescents improved the position of hyoid bone and enlarged airway dimensions [41, 42]. Similar to the outcomes of above research, we observed a significant rise in VPA, LPA, Min GCSA, Min LCSA, and Min UCSA. Moreover, the hyoid position changed in an anterior-inferior direction, which may relate to the airway length increasing during growth and devlopment [16, 43]. Moreover, the value of ΔVPA, ΔGPA and ΔLPA is significantly larger in teenagers than the amount in the adult groups. The research of Tanaka et al. [44] has suggested that the mandible will adapt forward in patients during growth and development, and the mandible may migrate more forward with the decrease of the inclination of OP plane [25, 44]. This study speculates that this mandibular advancement in adolescents leads to a more notable improvement in the upper airway following orthodontic treatment.

Craniocervical posture is correlated with the function of craniocervicomandibular system, and reflects the balance among post-cervical, suprahyoid, infrahyoid and masticatory muscle groups [33, 45]. And it is also reliant on different sitting and standing postures [46]. In comparison to standing, postural muscular activity may be lower during sitting, meanwhile, the cervical spine position is more susceptible to the thoraco-lumbar spine and obesity [46, 47]. Accordingly, to eliminate the potential effects of above variables, we selected the standing posture for our evaluation.

Recently, the interrelation between malocclusion and craniocervical posture causes concerns in orthodontic field [23, 26, 32, 48]. Numerous studies have shown significant correlations between craniocervical posture and craniofacial morphology [48–50]. Thus, we considered whether orthodontic effects on the craniofacial complex could improve craniocervical posture in patients with class II skeletal high angles. To our knowledge, no researches were performed to about that. In this study, a noticeable increase in craniofacial angulation and a decrease of SN/CVT in adolescents were identified, suggesting that posterior tipping of the head occurred after treatment. Moreover, the middle and lower segments of cervical column were be more upright with the increase of CVT/Ver and EVT/Ver, as indicated by the result. However, no significant difference was observed in the craniocervical angles in adult patients after treatment. This outcome may be explained by a remarkable increase of the upper airway dimensions and anterior displacement of the hyoid bone, thus leading to a compensatory elimination of the craniocervical posture in adolescents. As revealed by the above finding, orthodontic treatment can be effective in correcting the forward-inclined craniocervical posture in adolescent patients with hyperdivergent skeletal Class II malocclusion.

More research has placed a focus on connection between dentofacial deformities and craniocervical function (e.g., skeletal morphology, upper airway, and craniocervical posture), whereas the variations by different age groups have ben rarely investigated [51–53]. In this study, the correlation analysis was conducted on the two types of patients to gain more insights into the physiological variations in the upper airway and craniocervical posture of patients with skeletal class II high angle at different ages, which may allow for more tailored treatment decisions for each group of patients. Accordingly, these variables prior to orthodontic intervention were applied to correlation analysis in case of its interference. The teenage group's findings demonstrated that the craniofacial and craniocervical angle was negatively correlated with FMA. In contrast, the majority of recent investigations on the correlation between craniocervical posture and vertical skeletal pattern has suggested that patients with larger FMA typically have larger craniocervical angle [23, 27, 54]. A hypothesis was proposed that high angle adolescent patients with upper airway constriction will extend their entire cervical column instead of merely moving head forward to obtain enough airflow. The possible reason for this hypothesis is that a significant-degree extension of craniofacial and craniocervical positions cannot be achieved without impairing the horizontal visual axis in adolescences with high angle [55]. Besides, the data of this study indicated that the hyoid position parameter H-FHP was correlated with inclination of middle cervical column. Cervical extension, as a compensating strategy, may facilitate the moving of the hyoid bone from the posterior pharyngeal wall permit the release of the obstructed airways [55, 56]. Although the findings of this study support this hypothesis, it is uncertain whether these discrepancies in craniocervical posture are correlated with upper airway dimensions in adolescents. However, for adult patients, we found that craniocervical posture was significantly correlated with chin position and vertical growth pattern. Nevertheless, in patients with little growth potential, it is basically not expected to perform growth interventions. Together with the results in Table 4, we concluded that orthodontic intervention can hardly correct the craniocervical posture of adult patients. As in the study of adolescents, upper airway and craniocervical position did not show a correlation in adult patients.

This study may have had some potential restrictions. First, the sample size is relatively small to prevents it from drawing generalized conclusions. Moreover, the information of craniocervical posture is relatively constrained since acknowledged measurement parameters are few and can only be evaluated in two-dimension images. Also, soft tissues are not covered due to the technological constraints of CBCT, whose changes may affect the airway and craniocervical posture outcomes.

In conclusion, orthodontic treatment can improve facial profile of individuals with skeletal class II high-angle, while also enhancing their upper airway morphology and craniocervical posture, where adolescents and adults differ greatly and the former show a more favorable change in craniocervical functional environment. Thus, we should pay close attention to the upper airway and craniocervical posture of adolescents. Through early orthodontic intervention, we are supposed to reconstruct craniocervical physiological ventilation and postural balance, so as to promote the benign growth and development trend of children's multidisciplinary orientation.

## Data Availability

The datasets used and/or analysed during the current study are available from the corresponding author on reasonable request.

## Abbreviations

OSAHS: Obstructive sleep apnea hypopnea syndrome
CBCT: Cone-beam Computed Tomography
MIA: Micro-implant anchorage
